# Computational tools and workflows in metabolomics: An international survey highlights the opportunity for harmonisation through Galaxy

**DOI:** 10.1007/s11306-016-1147-x

**Published:** 2016-12-27

**Authors:** Ralf J. M. Weber, Thomas N. Lawson, Reza M. Salek, Timothy M. D. Ebbels, Robert C. Glen, Royston Goodacre, Julian L. Griffin, Kenneth Haug, Albert Koulman, Pablo Moreno, Markus Ralser, Christoph Steinbeck, Warwick B. Dunn, Mark R. Viant

**Affiliations:** 1 School of Biosciences, University of Birmingham, Birmingham, B15 2TT UK; 2 Phenome Centre Birmingham, University of Birmingham, Birmingham, B15 2TT UK; 3 European Molecular Biology Laboratory, European Bioinformatics Institute (EMBL-EBI), Wellcome Trust Genome Campus, Cambridge, CB10 1SD UK; 4 Computational and Systems Medicine, Department of Surgery and Cancer, Imperial College London, London, SW7 2AZ UK; 5 Centre for Molecular Informatics, Department of Chemistry, Lensfield Road, Cambridge, CB2 1EW UK; 6 Manchester Institute of Biotechnology and School of Chemistry, University of Manchester, Manchester, M1 7DN UK; 7 MRC Human Nutrition Research Unit, Cambridge, CB1 9NL UK; 8 The Department of Biochemistry, University of Cambridge, Cambridge, CB2 1GA UK; 9 The Francis Crick Institute, 1 Midland Road, NW1 1AT London, UK

## Introduction

Over the past decade, metabolomics has transformed into a valuable scientific discipline, conducting comprehensive and (semi-)quantitative investigations of metabolism. Consequently, metabolomics is increasingly used across a wide range of research fields from basic biology to applied disciplines such as medicine, toxicology, environment and agriculture. Its expansion has been accompanied by a significant increase in the number of computational tools available to process and analyse metabolomics data. Such tools can provide automated and standardised operational pipelines for data pre-processing (e.g. chromatographic deconvolution or normalisation of data), univariate and multivariate statistical analysis, metabolite annotation and metabolic network modeling including deep learning strategies to detect new biological processes. Computational workflows are often complex and include a variety of tools and resources (e.g. scripting-language and version dependencies). For metabolomics to achieve its full potential in both the basic and applied sciences the accessibility, reporting, reproducibility and overall harmonisation of such computational tools and resources must be improved significantly. Only then can there be confidence that results obtained in one laboratory can be reproduced in another laboratory elsewhere across the globe. The development of standardised and reproducible computational workflows provides one route to achieving closer harmonisation. This is a logical evolution following the work of the Metabolomics Standards Initiative where one working group aimed to establish minimal reporting requirements for data processing (Goodacre et al. 2007).

Galaxy is a widely used workflow platform that has helped to transform genomics research by increasing the accessibility of powerful data processing and analysis tools to non-bioinformaticians; i.e., to bench biologists (See “How to Build Bioinformatic Pipelines Using Galaxy” The Scientist, August 1st, 2016).^1^ Two Galaxy workflows have recently been reported for metabolomics (Davidson et al. 2016; Giacomoni et al. 2015), representing a significant step towards harmonised metabolomics workflows. Building upon this, we have formed a consortium of United Kingdom laboratories with a mission to continue these efforts and to create and maintain standardised Galaxy workflows to be used by the metabolomics community. We also seek to coordinate our efforts with international communities working on Galaxy-based metabolomics tools and environments, such as Workflow4Metabolomics in France (Giacomoni et al. 2015).

Our UK consortium has worked in collaboration with ELIXIR–UK and the Metabolomics Society to jointly conduct a community survey to:

1. Gauge the current knowledge of workflows in the international metabolomics community, in particular Galaxy workflows;
2. Determine what computational tools are currently being used and hence which are needed to be added to the Galaxy toolshed.

Here we report the results of the computational workflow questionnaire, from which we conclude that there is community wide support for the development of standardised and reproducible workflows for the growing international metabolomics community. By sharing the full results, we hope that others will conduct additional analyses and be able to derive further interesting conclusions, which will collectively help to drive more open and transparent approaches for data analysis and software development in metabolomics.

## Results questionnaire

We assembled a questionnaire comprised of 59 questions that asked for basic information about the respondent and their metabolomics experience as well as which software and hardware they used for their research. The questionnaire was conducted online using Survey Monkey (https://www.surveymonkey.com), and was open for about 12 weeks (from 16th November 2015 to 8th February 2016). It was promoted through multiple channels, including MetaboNews (http://www.metabonews.ca), websites (e.g. https://wiki.galaxyproject.org/GalaxyUpdates), metabolomics mailing lists, Twitter and metabolomics-related meetings and workshops. The majority of questions were multiple choice and respondents were able to select multiple answers for many of the questions (i.e. select all that apply). Respondents were guided to only answer questions related to their specific expertise, e.g. respondents who only use LC–MS were excluded from the subsequent questions about NMR spectroscopy. Here we summarise the most important findings. The complete set of responses (excluding personal identifiers) are available in full as electronic supplementary material (Supplementary Information 1–3 (SI1–3) and http://metabolomicssociety.org/computational-tools-and-workflows-questionnaire).

In total, 71 people responded to the survey, from which *ca.* 80% were academic scientists. No respondents were excluded from the survey. The respondents were at different stages of their academic career (such as PhD, postdoctoral researchers, scientific staff and principal investigators), which is also represented by their degree of experience in the field (2–4 years = 32%; 4–6 years = 20%; >8 years = 20%;). The majority (61%) of the respondent’s daily activities consisted of a combination of “wet” and “dry” laboratory work. In terms of geographical spread, the highest percentage of respondents were from France (32%) followed by Germany (12%), the UK (12%) and the USA (9%). Respondents used a large variety of analytical techniques (e.g. NMR spectroscopy and mass spectrometry) and applied metabolomics in different areas of science. Overall, the questionnaire included a relatively wide and diverse distribution of metabolomics practitioners (See SI 2, and [*Metabolomics Society website, url not available yet*]), suggesting that it fairly represents the community wide practices, though with a bias towards academic disciplines.

Respondents were then asked general questions regarding their use of workflows, if any, and other computational needs and resources as well as training needs in using workflows. Regarding bottlenecks in existing workflows, they stated that “data processing and statistical analyses” (68%) are the most time consuming steps in their workflows, followed by “Data curation” (45%). Also, approximately half of all respondents do not have access to dedicated bioinformatics support (51%). These findings reveal the need to develop accessible and reproducible tools and workflows that focus specifically on removing these main bottlenecks, without the need for skilled bioinformaticians (who are in short supply and high demand). In terms of their current knowledge of workflows, respondents are more aware of the Galaxy platform than any other workflow environment (59%). While the active use of (any) workflow platforms in metabolomics is still low (25% had no knowledge or experience), responders are using the Galaxy platform more than any other (Taverna—4%, KNIME—3% and Galaxy—13%). Approximately 99% of respondents stated that they would (51%) or possibly would (48%) use Galaxy, if their most used metabolomics tools were incorporated into that platform. Furthermore, a relative high percentage of respondents anticipate that their data would require access constraints (e.g. password protection or anonymisation) in any of the workflows developed (63%). In terms of education, respondents would (68%) or possibly would (31%) like to receive training in the operation of Galaxy metabolomics workflows. Respondents were also positive about receiving training in the development of Galaxy metabolomics workflows (59%—would and 32%—possibly would). In view of these encouraging findings, it is clear that bioinformatics in our community should build on the existing support of Galaxy through the development of Galaxy-specific tools as well as training courses for applied scientists, and hackathons for those wishing to further develop Galaxy tools and workflows.

Additional questions were asked regarding analytical and computational tools used to process, analyse and annotate metabolomics data. Approximately 74% of the responders typically conduct about five or more studies per year, and conduct either untargeted (87%) and/or targeted (52%) approaches. By far the majority of respondents utilised LC–MS (83%) methods in their research, followed by GC–MS (30%), NMR (26%), DIMS (16%), imaging mass spectrometry (6%, see (Palmer et al. 2016) for a specific survey on Imaging Mass Spectrometry), MALDI–MS (4%) and CE–MS (3%). It is worth noting that some of the respondents use multiple technologies across their studies. Encouragingly, a wide distribution of open data formats are used across the different MS (e.g. mzXML 70% and mzML 41%) and NMR platforms. Although mzML (Martens et al. 2011) and nmrML (http://www.nmrml.org) are the community recommended data standards, it is not clear from the survey that such acceptance and harmonisation exists. Considering that mzXML (Lin et al. 2005) is the precursor to mzML, adoption at this level is very encouraging. However, more community efforts are required to further reinforce the benefits of using established data standards (Salek et al. 2015; Rocca-Serra et al. 2016). The majority of respondents (91%) produce datasets that comprise of 50–500 samples. A small percentage (7%) produce datasets that consist of >1000 samples per study. The file sizes across those studies vary with the majority of those (86%) working with file size ≤250 Megabytes per sample. However, 14% were using files sizes >1 Gigabyte. The backend specifications of a workflow platform, such as Galaxy, should therefore be sufficient for uploading, analysing and storing what can potentially be a high number of large files. It is notable and arguably encouraging that *ca.* 41% of respondents have used open data repositories (e.g. MetaboLights or Metabolomics Workbench) in their research (Haug et al. 2012; Sud et al. 2016). This value is significantly larger than when the same question was asked in an international survey in 2014 (7%) (Weber et al. 2015). While this indicates the community is gradually recognising the value of such repositories (or perhaps are responding to the demands of journal editors or funding bodies), more needs to be done to further encourage the community to use repositories within standardised workflows.

The international survey, for the first time, also revealed the relative uses of multiple data processing software packages and tools (See SI2).

- Respondents that conduct LC–MS primarily used open source software (84%) and/or commercial software bundled with the instrument (65%); the open source software is strongly dominated by XCMS (70%), with mzMine and mzMine2 the next most utilised tools (26%).
- Respondents that conduct GC–MS are almost equally split between commercial software bundled with the instrument (76%) and open source software (67%). The commercial software is dominated by Agilent Chemstation (45%) while the non-commercial software is dominated by AMDIS (50%) and XCMS (40%).
- Respondents that conduct NMR spectroscopy primarily used commercial software bundled with the instrument (*ca.* 78%) and/or secondarily third-party commercial software (*ca.* 50%); the commercial software that is primarily utilised is Bruker’s TopSpin (56%) while the non-commercial software that is primarily utilised are NMRlab/MetaboLab (17%) and rNMR (17%).
- Respondents that conduct DIMS primarily used open source software (55%) although there are also a large number of responders using commercial software (bundled with the instrument 36% and/or third party 18%); the open source software is again dominated by XCMS (50%).

Based on these findings we believe that data-processing is a relatively well established process within MS and NMR-based metabolomics. While XCMS is the dominant solution to process MS data (i.e. LC–MS, GC–MS and DIMS), NMR data processing is mainly conducted using commercial software. Unfortunately, commercial software is largely unsuitable for implementing into freely available Galaxy workflows due to license issues. However, for both MS and NMR metabolomics methods, open-source data processing tools are available within Galaxy (Davidson et al. 2016; Giacomoni et al. 2015).

Further questions were asked about the types of data analyses that are currently being conducted in the community. The majority of respondents performed statistical analysis in their research (85%), and used a combination of univariate (e.g. Student t-test 91%; ANOVA 89%; Mann–Whitney U test 54%; Benjamini–Hochberg false discovery rate correction 50%; Kruskal Wallis 44%) and multivariate (e.g. PCA 96%; PLS-DA 73%; O-PLS 39%; HCA 39%; Random Forest 27%) methods (See SI2). Additionally, the majority of the respondents performed metabolic network or enrichment analyses (69%). The most popular software packages applied to perform this latter type of analysis were Cytoscape (41%) and MetaboAnalyst (55%). These high percentages confirm the importance of statistical and network-based tools in metabolomics research. Therefore, the full suite of common methods, as listed here, should be considered for implementation into Galaxy to address the needs of the community; good progress has already been achieved within the W4M Galaxy workflows (Giacomoni et al. 2015). However, poor experimental design and the misuse of statistics (e.g. underpowered experiments due to limited biological replication as well as the incorrect reporting of significance) can lead to the reporting of spurious and non-reproducible findings. For the community to benefit fully from the Galaxy implementation of these statistical methods, accompanying documentation and training should be developed and provided to ensure high quality metabolomics studies.

To complete the survey, one of the final steps in a typical metabolomics study was investigated (See SI2), that of metabolite annotation and/or identification. It is often regarded as the largest bottleneck in metabolomics research (Dunn et al. 2012), and there is an increasing requirement in metabolomics research to annotate tables of metabolites according to their level of identification. To provide a definitive identification of a metabolite, at least two independent orthogonal data that match an authentic compound measured under identical experimental conditions to the unknown compound are required (Sumner et al. 2007). Putatively annotating and characterising compounds from full-scan MS1 can still be very informative, however, and is widely used in metabolomics. This is highlighted in the survey as 80%, of the respondents that answered, actively conducted metabolite annotation using full scan MS1 data. Of those, 70% used CAMERA to “annotate” full-scan MS1 data (i.e. isotopes and adducts). Several other tools and software are used for MS1 annotation but each at a much lower percentage (<15%). The range of tools, databases and software packages used by the respondents to annotate MS^*n*^ data are more diverse and distributed; the top five included: Metlin 60%; MetFrag 45%; XCMS 44% and RMassBank 19%; NIST libraries and AMDIS 32% were used for GC–MS data annotation. For NMR Chenomx’s NMR suite (39%) and Bruker’s AMIX (39%) were the two main commercial software packages used to annotate, followed by open source solutions such as the Birmingham Metabolite Library and associated data mining tools (22%), BATMAN (17%), and rNMR (9%). The diversity of existing Galaxy tools used to annotate MS and NMR-based data is limited in comparison to other software tools used by the community. Therefore, the widely used open-source metabolite annotation software should be wrapped for use in Galaxy to allow comprehensive metabolomics analysis from initial data processing through to metabolite annotation.

## Conclusions from the survey

There is a need to develop tools as part of a user-friendly, automated data analysis workflow platform, such as Galaxy, which requires minimal bioinformatics skills to use, and that is well supported through community training (e.g. workshops, web-based tutorials and videos). The community is ready to accept such workflows.

Data processing workflows are relatively well established for MS and NMR-based metabolomics. For both, MS and NMR metabolomics, data processing tools are already available within Galaxy (Davidson et al. 2016; Giacomoni et al. 2015). Therefore, long term plans for implementation, growth, support and maintenance of those current Galaxy tools and workflows is recommended.

A large proportion of respondents use commercially-licensed, Windows- and GUI-based software for data processing and data analysis. This limits the adoption of such software within an open access and shareable workflow environment; where possible and necessary, open-source tools should be wrapped to provide an alternative via Galaxy. Additionally, the community should increase the efforts to involve commercial software partners and companies as part of our community-based workflow developments to further improve integration and interoperability of current non-compatible software within standardised (Galaxy) workflows.

Univariate and multivariate statistical tools are extensively used to analyse metabolomics data. All common statistical and network-based analysis tools, as indicated by respondents above and that are not already readily available in Galaxy, should be considered for implementation to fulfil the needs of the community, building on the progress already reported (Giacomoni et al. 2015).

The development of new analytical and computational methods for metabolite annotation is an active area of research in metabolomics. However, the number of tools to annotate and structurally identify metabolites within Galaxy is currently limited. Therefore, open-source metabolite annotation and identification software, which is widely-used by the community, should be prioritised for inclusion within Galaxy workflows.

While the use of data repositories has increased (in terms of the percentage of respondents using these commodities over the last *ca.* 2 years), efforts should continue in community education about the value of these resources, further training in their use, and scripts embedded in Galaxy to facilitate automated deposition and integration into standard workflows.

## Electronic supplementary material

Below is the link to the electronic supplementary material.

Supplementary material 1 (ZIP 67 KB)

Supplementary material 2 (ZIP 6 KB)

Supplementary material 3 (ZIP 32 KB)

## Footnotes

1 “How to Build Bioinformatic Pipelines Using Galaxy | The Scientist ...” 2016. 18 Aug. 2016 http://www.the-scientist.com/?articles.view/articleNo/46670/title/How-to-Build-Bioinformatic-Pipelines-Using-Galaxy/.
