# Supplementary material for: Computational tools and workflows in metabolomics: An international survey highlights the opportunity for harmonisation through Galaxy
Source: Metabolomics. 2016 Dec 27;13(2):12. doi: 10.1007/s11306-016-1147-x (PMC5192046; doi:10.1007/s11306-016-1147-x)
Supplement: Supplementary file 2 — Supplementary material 2 (ZIP 6 KB) [file 11306_2016_1147_MOESM2_ESM.zip › Suppl_02_Metabolomics_Survey_Workflows_and_tools_Dashboard.html]

:: Survey - Computational tools and workflows in metabolomics: What is our international community using? - Dashboard ::


## Computational tools and workflows in metabolomics (59 questions, respondents=71).Background Questions (subset) | | | | | | | | --- | --- | --- | --- | --- | --- | | **1. Area of Employment** **2. What type of work do you do?** | | **3. Country of respondent** | | **4. Job Position** **5. Years of Experience** | | | | | | | --- | --- | --- | | **6. In what area(s) of science are you currently applying metabolomics?** | **7. What type of analytical metabolomics experiments are you performing?** | **8. What type of bioinformatics are you currently performing?** | | | | | | | --- | --- | --- | --- | | | **9. What programming skill(s) and language(s) do you use?** | **10. What data repositories are you using?** | | --- Computational tools and workflows questions (subset) | | | | | --- | --- | --- | | | **1. What is your current knowledge of workflow platforms?** | | | | | | | | --- | --- | --- | --- | | **2. What NMR tools and softwares do you use for data processing?** | **3. What GC-MS tools and software do you use for data processing?** | **4. What LC-MS tools and softwares do you use for data processing?** | **5. What DIMS tools and softwares do you use for data processing?** | | | | | | --- | --- | --- | | **6. What methods do you apply for univariate statistical analysis?** | **7. What methods do you apply for multivariate statistical analysis?** | **8. What methods do you apply for metabolic network and/or enrichment analysis?** | | | | | | --- | --- | --- | | **9. What tools/algorithms/software do you use for metabolite annotation and/or identification applying full-scan MS data?** | **10. What tools/algorithms/software do you use for metabolite annotation and/or identification applying MSn data?** | **11. What tool/software/algorithm do you use for metabolite annotation and/or identification applying NMR data?** |
